# 

**Published:** 1897-06

**Authors:** 


### CONTENTS.

### COMMUNICATIONS.

Address........................................................................ 261
Experience in Dentistry........................................................ 265
Porcelain Inlays............................................................... 270
A Suggestion or Two ........................................................... 275
Judgment....................................................................... 278
Pyrozone—Its Uses ............................................................. 281

### SELECTIONS.

Incisions made in the Skin of the Face......................................... 264
The Degree of Anæsthesia Which Should be Induced Prior to Surgical Operations.................................................................... 285
New Developments in the Germ Theory of Disease................................ 286
Tobacco and Education ......................................................... 289
Artificial Camphor ............................................................ 289
The Science of the Morning Fast ............................................... 290
124 Years Old.................................................................. 290
Administration of Ether to Infants ............................................ 291
The Production of Aluminum in the United States................................ 291
Molding of the Superior Maxilla in Adenoid Vegetations......................... 292
The Spitting Animal ........................................................... 293
To Lessen the number of Dispensaries........................................... 293
Notes on Tuberculosis—Avenues of Invasion..................................... 294
Extracts from Dr. M. B. Campbell's Annual Report................................ 294
Are We Longer-Lived than Our Fathers?........................................... 295
Fetid Breath................................................................... 295
Hygienic Precepts.............................................................. 296
Board of Health Statistics Show Favor to Lady Bicyclers ........................ 296
Hygiene of the First Dentition ................................................ 297
Quinine ....................................................................... 297
Commercial Medical Colleges.................................................... 298
They Keep Their Hands Clean.................................................... 298
Buffet Cattle Cars .. ......................................................... 299
Get Your Pedigree Ready......................................................... 299
A Member of One of the Learned Professions ..................................... 300
How to Prescribe................................................................ 300
"How?" and "Why?"................................................................ 301
Insanity of English People ..................................................... 301
Pure Water ..................................................................... 302
Cremation or Burial?............................................................. 302
The Struggle for Life ........................................................... 303
Anecdote Regarding Lord Lister................................................... 303
The Science of Nutrition ........................................................ 304
A New Hæmostatic................................................................. 304
Nitrous-Oxide Gas with Oxygen.................................................... 305

### NOTICES.

New Jersey State Dental Society.................................................. 305
American Dental Association ..................................................... 306
The Indiana State Dental Meeting.................................................. 306

### EDITORIAL

To Old Point Comfort.............................................................. 307
Meetings of The American Dental Association and The Southern Dental Association......................................................... 308
Meeting of the National Association Dental Faculty ................................ 309
Biographical...................................................................... 310
Frank Abbott, M. D................................................................. 312

